# Shared decision making and chronic diseases

**DOI:** 10.1186/1710-1492-6-S4-A8

**Published:** 2010-12-10

**Authors:** Sophie Desroches

**Affiliations:** 1 Department of Food Sciences and Nutrition, Laval University, Québec, G1V 0A6, Canada

## 

Chronic diseases are the major cause of death and disability worldwide. Healthcare systems are reforming themselves to improve the health of people with, or at risk of developing, chronic diseases. One of the major goals of these health reforms is to provide both self-management support and decision support to patients so that they become involved and informed [1]. “Shared decision making” (SDM) is described as a process in which the health professional and patient go through all phases of the decision-making process together and in which they share the preference for treatment and reach an agreement on treatment choice [2]. It has been positioned between a paternalistic model, where the health professional assumes the leading role in treatment decision-making, and an informed patient choice model where the health professional’s role is limited to providing information and the patient is responsible for treatment decision-making [3].

SDM is recommended because of its potential to improve the quality of the decision-making process of patients for treatment choices that are informed and value-based, adherence to treatment decisions and ultimately, patient outcomes. It is one of the elements commonly considered important in patient-centered care, a gold standard to improve the quality of chronic disease care. Recently, SDM has also been advocated as a promising strategy to promote effective knowledge translation (KT) between patients and their health care provider. In that context, a SDM approach to KT is defined as a process that is embedded in a specific relationship and by which both the health care provider and the patient influence each other’s cognitions, emotions and behaviours, and come to agreement about a decision [4]. Although there is a growing clinical interest in SDM, it is still not widely adopted by health professionals.

Frequently reported barriers include time constraints, lack of applicability due to patient characteristics and lack of applicability related to the clinical situation. Otherwise, factors such as the health professional’s motivation, positive impact on the clinical process, and positive impact on patient outcomes are often reported as facilitating the adoption of SDM [5]. Theory-based approaches are suggested to better inform the design of SDM implementation interventions, as they provide insights on factors that may influence the adoption of SDM by health professionals and patients in a given context. In conclusion, SDM is increasingly regarded as an ideal chronic disease care strategy. SDM may enhance adherence to treatment decision, which is a major public health issue in chronic care.
